# Cytoplasmic Translocation of Polypyrimidine Tract-Binding Protein and Its Binding to Viral RNA during Japanese Encephalitis Virus Infection Inhibits Virus Replication

**DOI:** 10.1371/journal.pone.0114931

**Published:** 2014-12-29

**Authors:** Deepika Bhullar, Richa Jalodia, Manjula Kalia, Sudhanshu Vrati

**Affiliations:** 1 National Institute of Immunology, New Delhi, India; 2 Vaccine and Infectious Disease Research Centre, Translational Health Science & Technology Institute, Gurgaon, India; Wuhan University, China

## Abstract

Japanese encephalitis virus (JEV) has a single-stranded, positive-sense RNA genome containing a single open reading frame flanked by the 5′- and 3′-non-coding regions (NCRs). The virus genome replicates via a negative-sense RNA intermediate. The NCRs and their complementary sequences in the negative-sense RNA are the sites for assembly of the RNA replicase complex thereby regulating the RNA synthesis and virus replication. In this study, we show that the 55-kDa polypyrimidine tract-binding protein (PTB) interacts *in vitro* with both the 5′-NCR of the positive-sense genomic RNA - 5NCR(+), and its complementary sequence in the negative-sense replication intermediate RNA - 3NCR(-). The interaction of viral RNA with PTB was validated in infected cells by JEV RNA co-immunoprecipitation and JEV RNA-PTB colocalization experiments. Interestingly, we observed phosphorylation-coupled translocation of nuclear PTB to cytoplasmic foci that co-localized with JEV RNA early during JEV infection. Our studies employing the PTB silencing and over-expression in cultured cells established an inhibitory role of PTB in JEV replication. Using RNA-protein binding assay we show that PTB competitively inhibits association of JEV 3NCR(-) RNA with viral RNA-dependent RNA polymerase (NS5 protein), an event required for the synthesis of the plus-sense genomic RNA. cAMP is known to promote the Protein kinase A (PKA)-mediated PTB phosphorylation. We show that cells treated with a cAMP analogue had an enhanced level of phosphorylated PTB in the cytoplasm and a significantly suppressed JEV replication. Data presented here show a novel, cAMP-induced, PTB-mediated, innate host response that could effectively suppress JEV replication in mammalian cells.

## Introduction

The *Flavivirus* genus of the *Flaviviridae* family of animal viruses contains more than 70 viruses including medically important dengue virus (DENV), tick-borne encephalitis virus (TBEV), West Nile virus (WNV), Yellow fever virus (YFV) and Japanese encephalitis virus (JEV). JEV is responsible for frequent epidemics of encephalitis in humans in most parts of Southeast Asia, China, Korea, Japan, and India. It is a neurotropic virus accounting for ∼50,000 cases of encephalitis annually of which ∼30% result in mortality and another ∼30% in long lasting neuropsychiatric complications [1]. The treatment strategies upon diagnosis of JEV infections are mostly supportive and symptomatic as no specific therapeutic treatment is presently available. Greater understanding of the molecular mechanisms controlling JEV replication could help in designing novel interventions.

JEV has a single-stranded positive-sense RNA genome encased in the nucleocapsid surrounded by membrane envelope containing structural proteins. The genomic RNA has a type I cap (m7GpppAmp) at the 5′-end and lacks polyadenylation at its 3′-end. The ∼11-kb genome has a single open reading frame (ORF) encoding a polyprotein of 3432 amino acids that is subsequently cleaved to produce three structural proteins, capsid (C), pre-membrane (prM) and envelope (E), and seven non-structural proteins, NS1, NS2A, NS2B, NS3, NS4A, NS4B and NS5 (REF). The ORF is flanked by 95- and 585-nucleotides long non-coding regions (NCRs) at the 5′- and 3′-ends, respectively [2]. The 3′ distal region of ∼100 nucleotides within the 3′-NCR is predicted to form a stable stem loop (SL). Although the size and the nucleotide sequence of the NCRs differ among different flaviviruses these sequences adopt a secondary structure of similar size, shape and predicted thermodynamic stability [3], [4]. The conservation of RNA structures and their location among flaviviruses in the 5′- and 3′-NCRs suggest their possible functional relevance in viral replication [5], [6].

Following the infection, the positive-strand JEV genomic RNA is released from endocytosed virions into the cytoplasm of the infected cell initiating the replication and synthesis of viral proteins. The positive-sense genome is transcribed into the negative-sense RNA replication intermediate (antigenome) which is then used as a template for the synthesis of a large number of copies of the positive-sense genomic RNA. The genome replication involves its circularization that is mediated by long-range RNA-RNA interactions between sequences from 5′- and 3′-NCR [7]. The promoter for DENV RNA synthesis is a large SL structure located in the 5′-NCR to which the replicase complex containing the RNA-dependent RNA polymerase (RdRp) protein NS5 binds in association with the viral protease/helicase protein NS3, other viral non-structural proteins and presumably host factors [5], [8].

A number of host proteins have been identified that interact specifically with NCRs of genome and/or complementary antigenome regions of the different flaviviruses. Interestingly, most of these host proteins have been shown to regulate the viral replication process either positively or negatively. Thus, interaction of TIA-1 and TIAR proteins with WNV antigenome [9], La protein with JEV genome [10], [11], NF90/NFAR group of proteins with Bovine viral diarrhoea virus genome [12], and polypyrimidine tract-binding protein (PTB) with Dengue virus genome [13]–[15] was required for efficient viral replication. On the other hand, there is a growing list of host proteins being identified to negatively regulate viral replication. For example, Hsp40 chaperone protein DNAJC14 inhibited YFV replication [16], FUSE binding protein 1 (FBP1) inhibited JEV replication [17], and Y-box binding protein 1 (YB-1) inhibited DENV type 2 (DENV-2) replication [18].

PTB belongs to an hnRNP1 family of RNA-binding proteins [19] and is involved in several aspects of cellular mRNA metabolism including splicing, RNA stability, and internal ribosome entry site (IRES)-mediated translation of viral and cellular mRNAs [20]–[22]. Our understanding of the role of PTB in virus replication is largely based on studies with hepatitis C virus (HCV) and picornaviruses where it is involved in IRES-mediated translation initiation [23]–[27]. In addition, PTB was shown to bind the 3′-NCR of DENV-4 genome [14], and both 5′- and 3′-NCRs of Coxsackie virus B3 (CVB3) RNA [27], and the protein was important for efficient virus replication [13], [28]. PTB has also been shown to bind the 3′-NCR of JEV antigenome [3NCR(-)] [29] although its role in virus replication was not known. In the present study we demonstrate that PTB, in addition to binding JEV 3NCR(-) RNA, also binds 5′-NCR of JEV positive-strand RNA [5NCR(+)] *in vitro*. We also show that nuclear PTB relocalized to cytoplasm during JEV infection where it associated with viral RNA leading to inhibition of RNA transcription and virus replication. This constitutes a novel cellular mechanism employed by the host to control JEV infection.

## Materials and Methods

### Virus and cells

The P20778 strain of JEV was grown in porcine stable kidney (PS) cells and titrated on PS cell monolayers by plaque assay [30]. Human embryonic kidney 293 (HEK), African green monkey Kidney (Vero), and PS cells obtained from the National Centre for Cell Sciences, Pune were grown in DMEM (Gibco), EMEM (Sigma) and MEM (HyClone), respectively, supplemented with glutamine, 10% fetal calf serum, and antibiotic/antimycotic at 37°C with 5% CO_2_. HEK and Vero cells were transfected with plasmid DNA using Effectene (Qiagen) and JetPrime (Polyplus Transfection) reagents, respectively, as per the manufacturers' instructions. The nuclear and cytoplasmic extracts from cultured cells were prepared using NE-PER nuclear and cytoplasmic extraction reagent kit (Thermo Scientific).

### RNA synthesis *in vitro*

The RNA transcripts used to study RNA-protein interactions were generated by *in vitro* transcription using plasmids containing the cDNAs corresponding to JEV NCR sequences cloned under the control of bacteriophage T7 promoter. Plasmids pJE5NCR and pJE3SL, containing the JEV 5′-NCR cDNA in ApaI-BamHI sites and 3′-SL cDNA in ApaI-XbaI sites, respectively, have been described previously [11], [31]. pJE5NCR had JEV 5′-NCR sequence corresponding to nucleotides 1–95 whereas pJE3SL had JEV 3′-NCR sequence corresponding to nucleotides 10891–10976 of JEV plus-strand genome (GenBank accession no. NC_001437). Plasmid pJE3NCR(-) had JEV sequence corresponding to nucleotides 10882–10976 of the JEV minus-strand RNA in ApaI-XbaI sites. This sequence was complementary to nucleotides 1–85 of JEV 5′-NCR. For producing the radiolabelled transcript, pJE5NCR was linearized with BamHI, and pJE3SL and pJE3NCR(-) were linearized with XbaI, and the DNA purified using QIAquick Gel Extraction kit (Qiagen). Transcription was performed *in vitro* at 37°C for 1 h in a 20 µl reaction containing 40 mM Tris-Cl (pH 7.5), 6 mM MgCl_2_, 10 mM NaCl, 2 mM spermidine, 10 mM dithiothreitol (DTT), 0.5 mM each of ribonucleotides (A, C and G), 12 µM UTP (Promega), 50 µCi (^32^P)-UTP (3000 Ci/mmol, NEN), 1 µg linearized DNA template, 20 U RNasin (Promega) and 20 U T7 RNA polymerase (Promega). The reaction product was then treated with 10 U RNase-free DNase (Promega) for 20 min at 37°C. The transcript was then treated with phenol-chloroform, precipitated with ethanol, and resuspended in 20 µl RNase-free water. RNA yield was determined by the trichloroacetic acid (TCA) precipitation. A specific activity of ∼10^8^ cpm/µg was routinely obtained.

### Purification of recombinant proteins

*E. coli* BL21 (DE3) cells were transformed with the expression vector pET28a-PTB (a generous gift from Dr. J.G. Patton) and pET28a-NS5 (a generous gift from Dr. R. Chang), and the expression of his-tagged recombinant PTB or JEV NS5, respectively, was induced by 1 mM IPTG. The recombinant protein was purified using Ni^2+^-nitrilotriacetic acid-agarose (Qiagen) under non-denaturing conditions and eluted with 250 mM imidazole. The protein was electrophoresed on a 10% Sodium dodecyl sulphate (SDS)-poly acrylamide gel (PAGE) to confirm the size and the purity. Protein concentration was determined by the BCA method and the aliquots were snap frozen and stored at −70°C. Freshly-thawed protein aliquot was used for all assays.

### Electrophoretic Mobility Shift Assay (EMSA)

Purified recombinant PTB was incubated in binding buffer (14 mM HEPES pH 7.5, 6 mM Tris-HCl pH 7.5, 1 mM EDTA, 1 mM DTT, 60 mM KCl) with 500 ng yeast t-RNA and 1 U RNasin in a 20 µl reaction for 10 min at 30°C followed by the addition of 1 ng ^32^P-labelled RNA and incubation for 30 min at 30°C. The RNA-protein complexes were resolved by non-denaturing 6% polyacrylamide gel (PAGE) (acrylamide: bisacrylamide 50: 1) containing 2.5% glycerol in 0.5X TBE buffer (45 mM Tris-borate, 1 mM EDTA) at 4°C. The gels were autoradiographed after drying.

### Filter-binding assay

Increasing amounts of PTB were incubated at 30°C for 15 min in a 20 ml reaction with 100 fmol ^32^P-labelled RNA in binding buffer (14 mM HEPES, pH 7.5, 1 mM EDTA, 1 mM DTT, 60 mM KCl) containing 500 ng yeast tRNA. The reaction products were loaded onto nitrocellulose filters (HAWP 02500; Millipore) pre-equilibrated with 2 ml binding buffer, and filtered under vacuum. The filters were then washed twice with 2 ml binding buffer and dried, and the counts retained were measured in a liquid scintillation counter. The percentage of bound RNA was calculated from the counts and a saturation binding curve was plotted. The binding affinity of the RNA–protein complex, denoted as the dissociation constant (*K*_d_) of the binding reaction, was calculated as the protein concentration at which 50% of the RNA was bound. This was carried out by fitting the data into the binding curve (Langmuir) equation using GraphPad Prism software.

### Ultra-violet (UV) light induced cross-linking of RNA and protein

The RNA-protein binding reaction was set up as described above. After incubation for 30 min at room temperature, the binding reaction mixture was transferred to an ice bath and irradiated with 254-nm UV lamp (4 Watts) held at a 3 cm distance from the reaction mixture for 30 min. After irradiation, RNase A (20 µg) was added and the reaction mixture was incubated for 30 min at 37°C to digest the unprotected RNA. The UV cross-linked products were boiled in Laemmli sample buffer (0.125 M Tris-Cl, pH 6.8, 2% SDS, 5% β-mercaptoethanol, 10% glycerol, and 0.001% bromophenol blue) for 5 min at 100°C. The samples were electrophoresed on a discontinuous 6% sodium dodecyl sulphate (SDS)-PAGE. The gel was fixed in 7% acetic acid, dried and autoradiographed.

### RNA Co-immunoprecipitation and reverse transcription polymerase chain reaction (Co-IP RT-PCR)

Cells infected with JEV at a multiplicity of infection (moi) of 10 were harvested, washed twice with ice-cold phosphate-buffered saline (PBS), and collected in 500 µl cell lysis buffer A (10 mM HEPES, pH 7.0, 100 mM KCl, 5 mM MgCl_2_, 1 mM DTT, 2 mM phenylmethylsulfonyl fluoride, 100 µg/ml aprotinin and 0.5% Nonidet P-40). RNasin (Promega) and Protease inhibitor mixture (Roche) were added freshly. Cells were lysed by incubating on ice for 20 min with frequent pipetting up and down. The lysate was then centrifuged at 16,000 g for 15 min at 4°C and the supernatant collected. Protein G sepharose beads (10 µl) (Amersham) were washed with PBS, blocked with 2% bovine serum albumin (BSA) and incubated for 1 h with PTB (Abcam) or luciferase (Promega) antibody. Beads were treated with buffer B (50 mM Tris, pH 7.4, 150 mM NaCl, 1 mM MgCl_2_, 0.05% Nonidet P-40) and subsequently resuspended in 850 µl buffer B, 40 µl 0.5 M EDTA, 150 units RNasin (Promega) and 100 µl lysate. The reaction tubes were incubated on an end-to-end rotor overnight at 4°C. Beads were then washed thoroughly with ice-cold buffer B and resuspended in 100 µl buffer B supplemented with 0.5% SDS and incubated at 37°C for 30 min. RNA was extracted using the TRIzol reagent (Invitrogen). The RNA pellet resuspended in diethyl pyrocarbonate (DEPC)-treated water was used as the template for RT-PCR using JEV-specific oligonucleotide primers (Forward primer: 5′-AGCCATCGTGGTGGAGTA-3′; Reverse primer: 5′-GGTACCTTTGTGCCAATGGTGGTT-3′) that produce a 397-bp amplicon containing JEV genomic sequence between nucleotides 96–476 (GenBank accession no. NC_001437).

### Protein and RNA localization in infected cells

Cells grown in BD BioCoat Poly-L-Lysine-coated 35-mm culture dishes were infected with JEV or mock-infected. Cells were fixed by 2% paraformaldehyde and permeabilized with 0.5% saponin. Cells were incubated with 1% BSA followed by goat PTB antibody (Abcam) and mouse dsRNA antibody (English & Scientific Consulting Bt.). Following a wash with 1% BSA the cells were incubated with rabbit anti-goat Cy5 (Invitrogen) and donkey anti-mouse Alexa 488 (Invitrogen) secondary antibodies. The cells were washed again with 1% BSA and overlaid with mounting medium containing DAPI (Invitrogen) to stain the nucleus. Cells were visualized by Olympus Fluoview FV1000 microscope and images analysed using Olympus Fluoview Ver 2.1c software.

### PTB gene silencing and over-expression in mammalian cells

A plasmid encoding PTB shRNA was used for down-regulating the PTB level by targeted gene silencing in cultured cells. The PTB shRNA-encoding DNA was inserted between the BamHI-EcoRI sites in the RNAi-Ready pSIREN-RetroQ-ZsGreen vector (Invitrogen). The DNA encoding the shRNA was synthetically made by annealing the following oligonucleotides: 5′GGATCCGTGACAAGAGCCGTGACTACTTCAAGAGAGTAGTCACGGCTCTTGTCATTTTTTGAATTCTCTAGA-3′ (top strand); 5′-TCTAGAGAATTCAAAAAATGACAAGAGCCGTGACTACTCTCTTGAAGTAGTCACGGCTCTTGTCACGGATCC-3′ (bottom strand). A shRNA directed against luciferase gene (Clontech) was used as a negative control. For PTB over-expression, cells were transfected with pcPTB that was produced by cloning the PTB cDNA in pcDNA 3.1(-) vector (Invitrogen) under the control of CMV promoter. Plasmid pcDNA 3.1(-) vector was used as a negative control. Levels of PTB in transfected cells were measured by immunoblotting the cell lysate with PTB antibody.

### Real-time reverse transcription polymerase chain reaction

RNA from JEV-infected cells was isolated using TRIzol reagent (Invitrogen) and used for cDNA synthesis employing iScript cDNA synthesis kit (Bio-Rad) and primers specific for JEV positive (5′-AATAGGTTGTAGTTGGGCACTCTG-3′) and negative strand RNA (5′-AGAGCACCAAGGGAATGAAATAGT-3′). Maxima SYBR Green qPCR Master Mix (Thermo Scientific) was then used for quantitative real-time PCR using these primers [32]. For normalizing the JEV RNA amounts, the 18S ribosomal RNA was quantified using the following primers: Forward, 5′-CGAAAGCATTTGCCAAGAAT-3′; Reverse, 5′-AGTCGGCATCGTTTATGGTC-3′.

## Results

### PTB binds JEV NCR RNAs *in vitro*

**Figure 1:**
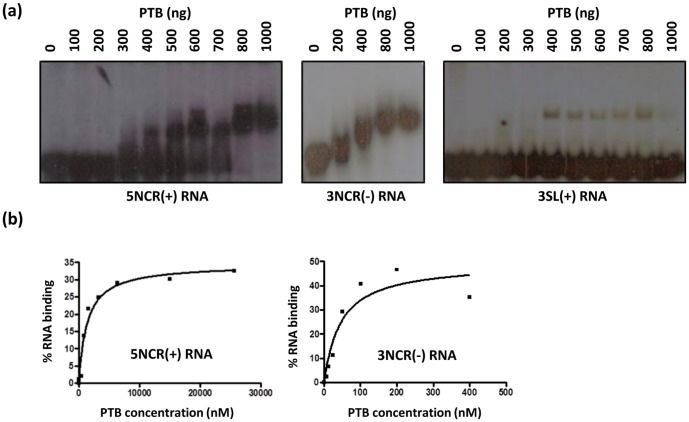
PTB binding with JEV RNA *in vitro*. (a) Binding reactions were carried out *in vitro* using 1 ng radiolabelled JEV RNA (indicated at the bottom) and increasing amounts of recombinant PTB (amounts in ng shown at the top). The RNA-protein complexes were resolved on a 6% non-denaturing PAGE followed by autoradiography. (b) Filter binding assays were carried out by incubating increasing amounts of PTB with 100 fmol ^32^P-labelled RNA in binding buffer containing 500 ng yeast tRNA. The percentage of bound RNA was determined and saturation binding curve plotted to calculate the dissociation constant (*K*_d_) of the binding reaction.

Radiolabelled 5NCR(+) and 3NCR(-) RNAs were incubated with increasing amounts of recombinant PTB in an EMSA in presence of 60 mM KCl to study possible RNA-protein interaction *in vitro*. Distinct retarded bands of radiolabelled RNA in RNA-protein complexes were observed on a native gel indicating that PTB bound to JEV 5′-NCR of the genomic RNA - 5NCR(+), and the corresponding complementary sequence in the antigenome - 3NCR(-) (Fig. 1a). These complexes were stable even in the presence of as high as 300 mM KCl in the binding reaction. The radiolabelled complex formation was inhibited when increasing amounts of corresponding cold RNA was added to the binding reaction (data not shown). We also tested binding of PTB to 3SL(+) RNA as a negative control where a weak band of RNA-protein complex was seen in presence of 60 mM KCl. This band showed no increase in intensity when PTB concentration in the binding reaction was increased. Additionally, this complex disappeared in presence of a higher salt concentration of 100 mM KCl (data not shown) suggesting a weak and non-specific interaction of PTB with 3SL(+) RNA.

The binding affinity of JEV RNAs with PTB was determined as the *K*_d_ of the binding reaction using filter-binding assays (Fig. 1b). Thus, the mean *K*_d_ for 5NCR(+) RNA binding with PTB was 1362 nM, whereas it was 41 nM for the 3NCR(-) RNA. These experiments thus show that PTB binding to JEV 5NCR(+) and 3NCR(-) was strong and specific, and the protein had higher affinity to 3NCR(-) RNA compared to 5NCR(+) RNA.

### PTB associates with JEV RNA in virus-infected cells

**Figure 2:**
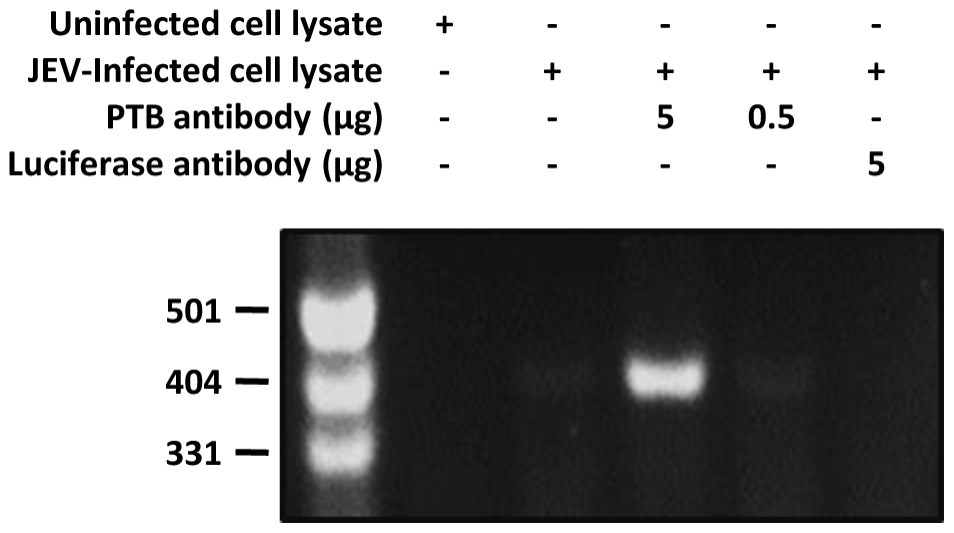
PTB binding with JEV RNA *in vivo*. HEK cells were infected with JEV at a moi of 10. Cell lysate prepared 24 h pi was immunoprecipitated using PTB antibody. A control immunoprecipitation reaction was carried out using Luciferase antibody. RNA extracted from the immunoprecipitated pellet was subjected to RT-PCR using JEV genome-specific primers. The RT-PCR products were electrophoresed on a 2% agarose gel along with DNA size markers (in bp) indicated at the left.

PTB interaction with JEV RNA during the course of the virus replication in the mammalian cell was studied by the Co-IP RT-PCR. JEV-infected HEK cells were lysed at 24 h post-infection (pi) and PTB-bound RNA precipitated using PTB antibody. RT-PCR of the precipitated RNA is expected to yield a 397-bp product in case it contained JEV RNA. A distinct band of expected size in the RT-PCR product was seen when PTB antibody was used for immunoprecipitation (Fig. 2). No amplification product was seen in the control reaction where luciferase antibody was used. While this experiment shows that JEV RNA could be interacting with PTB *in vivo*, it does not necessarily indicate a direct binding of the two. However, in light of direct RNA-protein binding *in vitro* (shown above) it may be inferred that PTB protein associated with JEV RNA during the virus infection of the cell. More evidence to this effect was provided by the experiment described below.

### PTB relocates to cytoplasm during JEV infection and associates with JEV RNA

**Figure 3:**
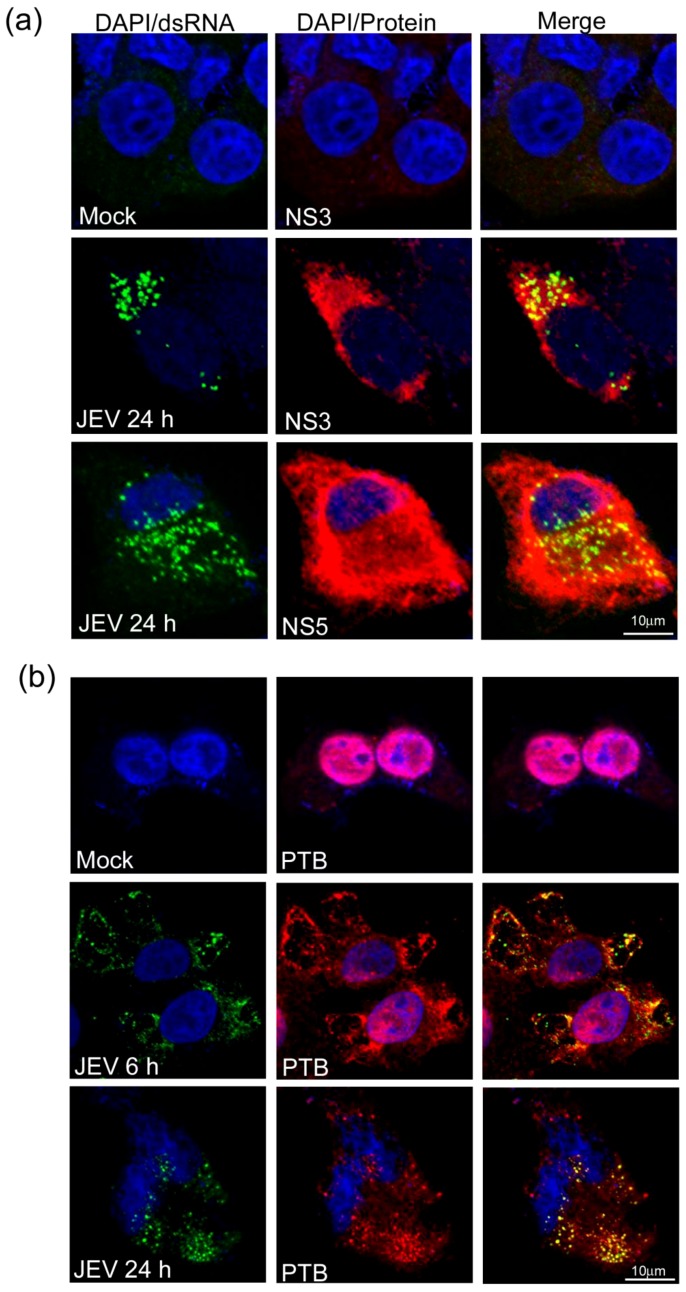
PTB colocalization with dsRNA in JEV-infected cells. Monolayers of Vero cells were infected with JEV at a moi of 10. At the indicated time pi, the cells were fixed, permeabilized, and stained with mouse dsRNA antibody, rabbit NS3 and NS5 antibodies (panel a), goat PTB antibody (panel b), followed by staining with anti-goat Alexa 568, anti-mouse Alexa 488, and anti-rabbit Alex 568 secondary antibodies. Cells were visualized after adding mounting medium with DAPI to stain the nuclei. Localization of dsRNA is shown with green fluorescence whereas PTB, NS3 and NS5 are shown with red fluorescence. Colocalization of dsRNA with PTB results in yellow fluorescence as seen in the merge panels.

Flavivirus replication occurs in replication complexes located on the membranous compartments of the endoplasmic-reticulum also known as vesicle packets. Several of viral non-structural proteins and the replicative form of the viral genome as the dsRNA have been shown to colocalize in these replication complexes [33]–[36]. Anti-dsRNA antibodies have been widely used to localize the flavivirus replication complex [37]–[39]. We studied the distribution of dsRNA and JEV non-structural proteins in JEV-infected Vero cells by confocal microscopy. The dsRNA could be stained in the cytoplasm of virus-infected cells and it strongly colocalized with JEV non-structural proteins (Fig. 3a). The Pearson Coefficient for dsRNA colocalization with NS3 and NS5 was 0.50 and 0.41, respectively.

PTB is located mainly in the nucleus in association with hnRNA. We studied the location of PTB during JEV infection and its localization with respect to JEV RNA by staining with dsRNA antibodies. In mock-infected Vero cells, PTB was located mostly in the nucleus and no dsRNA could be seen in the cell (Fig. 3b). In the JEV-infected cells, PTB could be seen in the cytoplasm as early as 6 h pi (Fig. 3b). The dsRNA representing JEV replication foci were distinctly seen in the cytoplasm at this stage and it was found to colocalize with PTB (Pearson Coefficient 0.39). More of PTB was seen in the cytoplasm at 24 h pi where it again showed colocalization with PTB (Pearson Coefficient 0.55). These data demonstrate that PTB migrates to the cytoplasm in JEV-infected cells where it colocalizes with dsRNA representing JEV replication. Similar findings were made in HEK cells also (data not shown).

**Figure 4:**
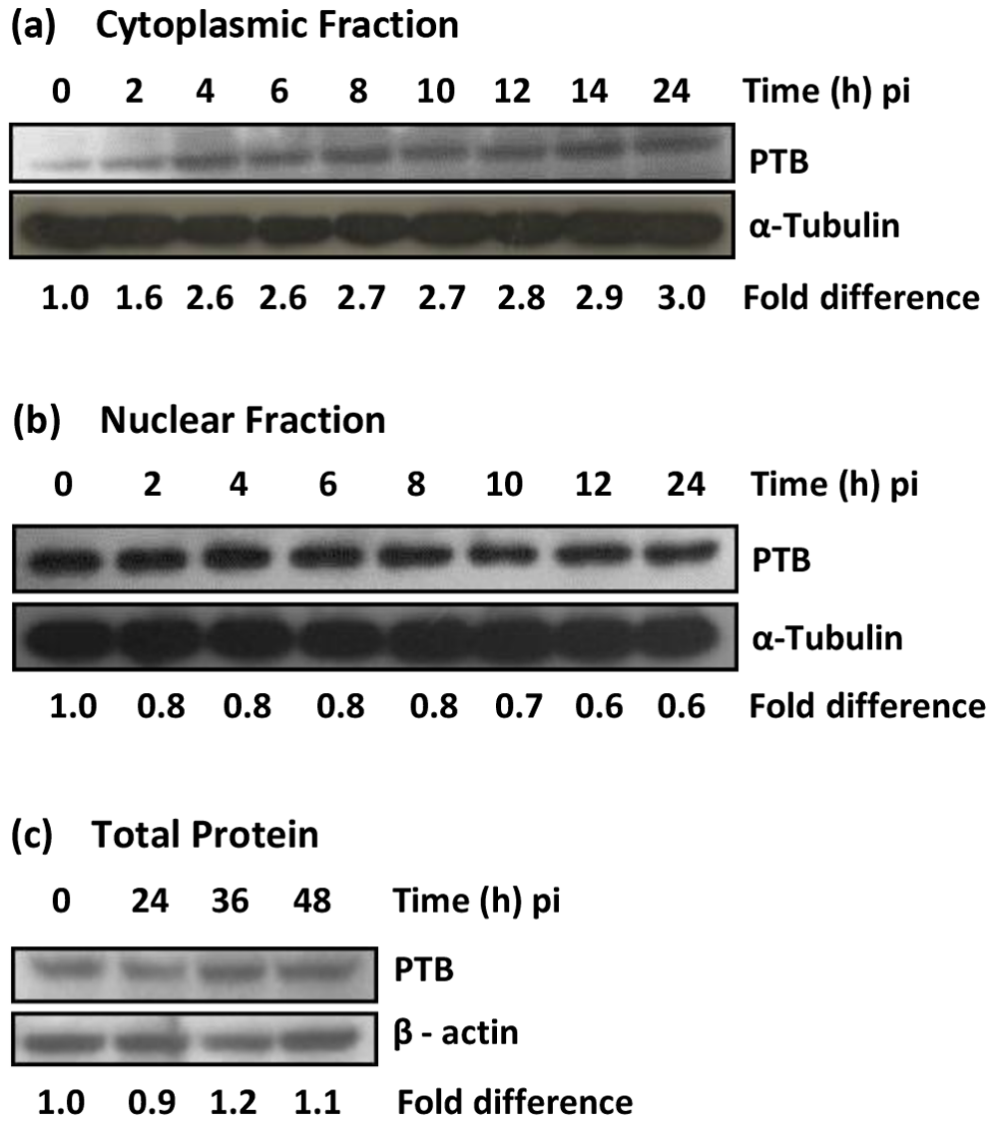
Analyses of cytoplasmic, nuclear and total PTB level in JEV-infected cells. Monolayers of Vero cells were infected with JEV at a moi of 5 and cells were harvested at different times pi. Proteins from various fractions were Western-blotted with PTB antibody and changes in PTB levels estimated based on the intensity of the bands in comparison to that seen at 0 h pi. α-Tubulin or β-actin were used as the loading controls. The figure shows PTB levels in (a) the cytoplasmic fraction, (b) the nuclear fraction, and (c) the total cell lysate prepared from JEV-infected cells.

To corroborate the above findings, nuclear and cytoplasmic extracts were prepared from JEV-infected Vero cells at various times pi using NE-PER Nuclear and Cytoplasmic Extraction Kit (Pierce) and the purity of the fractions established by Western blotting with specific antibodies. Before the virus infection, PTB was mainly in nuclear extract and only a small amount of it was detected in the cytoplasm (Fig. 4a). As the infection progressed, the levels of PTB progressively increased in the cytoplasmic fraction and at 24 h pi these were 3-folds higher than that seen at 0 h pi. Concomitantly, there was a progressive reduction in the PTB levels in the nuclear fraction and at 24 h pi it was 0.6-fold compared to that seen at 0 h pi (Fig. 4b). There was no significant difference in the total PTB levels during the course of JEV infection (Fig. 4c). That cells were indeed infected with JEV was established by blotting the lysates with JEV NS1 antibody (data not shown). These data demonstrated quantitative translocation of PTB from nucleus to cytoplasm upon JEV infection of Vero cells.

### PTB knock-down enhances JEV replication

**Figure 5:**
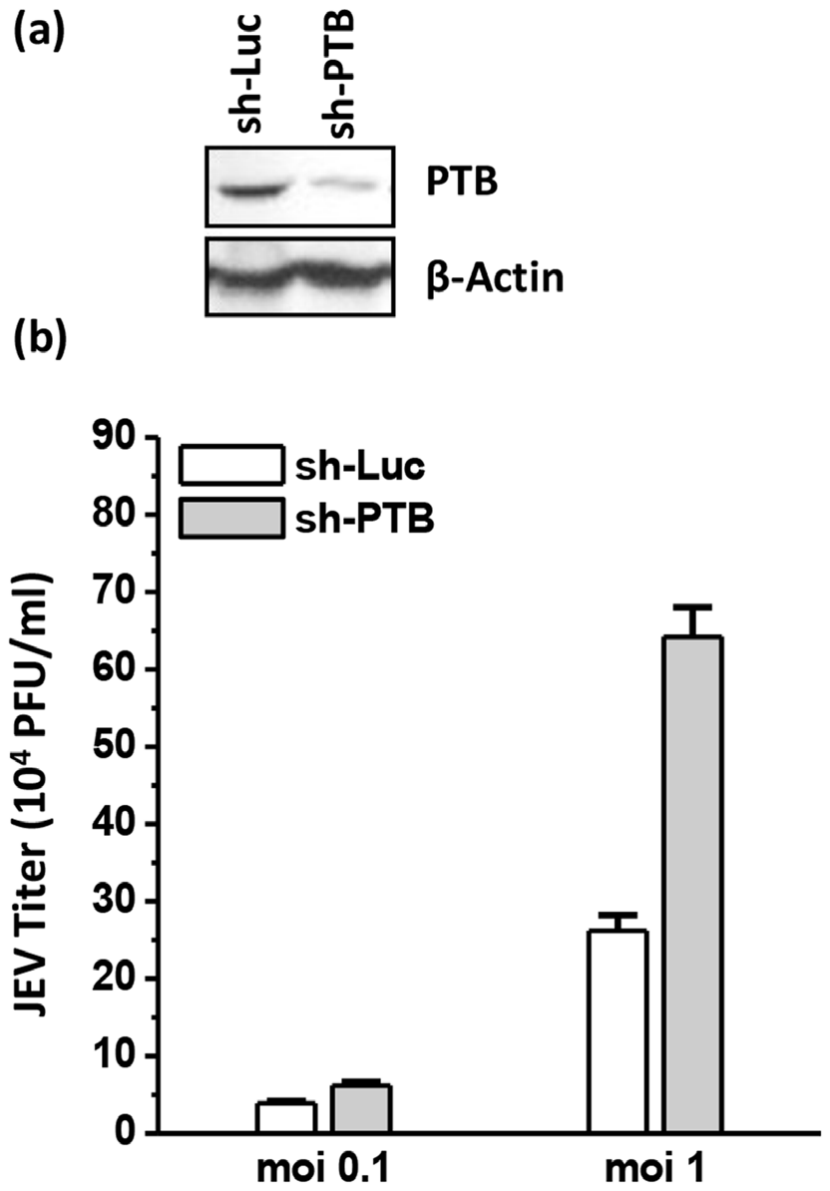
Effect of PTB knock-down on JEV replication in cultured cells. (a) Vero cells were transfected with plasmid encoding shRNA against PTB (sh-PTB) or Luciferase (sh-Luc) and analysed 48 h later by Western blotting for PTB levels. (b) Vero cells transfected with shRNA-encoding plasmids were infected 43 h later with JEV at a moi of 0.1 and 1. The culture supernatants were harvested 24 h pi and JEV titers determined. Mean virus titers and standard deviation from the experiment done in triplicates are shown.

In order to determine the role of PTB in JEV life cycle, the expression of PTB was knocked-down in cultured mammalian cells using plasmid expressing PTB-specific shRNA (sh-PTB). An shRNA against luciferase (sh-Luc) was used as a negative control. The level of PTB in Vero cells was reduced significantly 48 h after transfection with sh-PTB (Fig. 5a). The sh-PTB- or sh-Luc-transfected Vero cells were infected 43 h later with JEV and viral titers determined 24 h pi. Compared to sh-Luc-transfected cells, there was a small but consistent increase in JEV titers in PTB-silenced cells (Fig. 5b). Thus, titers were 60% and 150% higher in PTB knocked-down cells infected at a moi of 0.1 and 1.0, respectively. This effect was reproduced in HEK cells (data not shown). These data suggested an inhibitory role of PTB in JEV replication.

### PTB over-expression suppresses JEV replication

**Figure 6:**
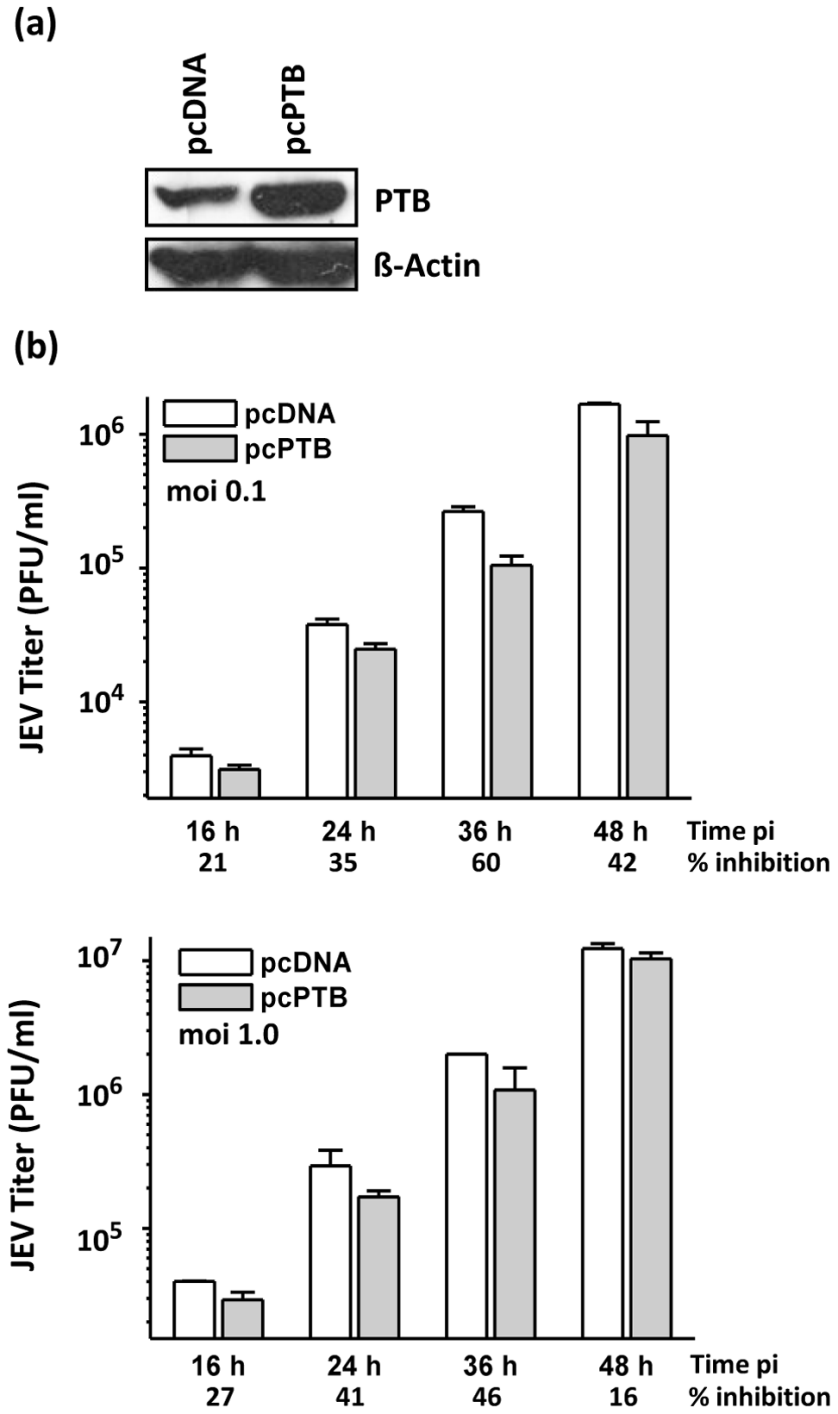
Effect of PTB over-expression on JEV replication in Vero cells. (a) Vero cells were transfected with pcPTB and levels of PTB checked by Western blotting 48 h later. (b) Vero cells transfected with pcPTB or pcDNA were infected 43 h later with JEV at a moi of 0.1 and 1. The culture supernatants were harvested at different time points and JEV titers determined. Mean virus titers and standard deviation from the experiment done in triplicates are shown. JEV titers in PTB over-expressing cells were consistently inhibited as indicated at the bottom of the figures.

To further confirm the inhibitory role of PTB in JEV replication, PTB was over-expressed by transfecting Vero cells with PTB-expressing plasmid pcPTB and viral titers determined. Western blotting showed a significant increase in PTB levels in pcPTB-transfected cells 48 h later (Fig. 6a). Vero cells infected with JEV 43 h after transfection with pcPTB showed small but consistent reduction in JEV titers (Fig. 6b). Compared to the control cells, PTB over-expressing cells showed 21–60% reduction in JEV titers at moi of 0.1 and 16–46% reduction in JEV titers at moi of 1.0 at different time points. These PTB knock-down and over-expression experiments together suggest an inhibitory role of PTB in JEV replication in mammalian cells.

### PTB affects viral RNA and protein levels in JEV-infected cells

**Figure 7:**
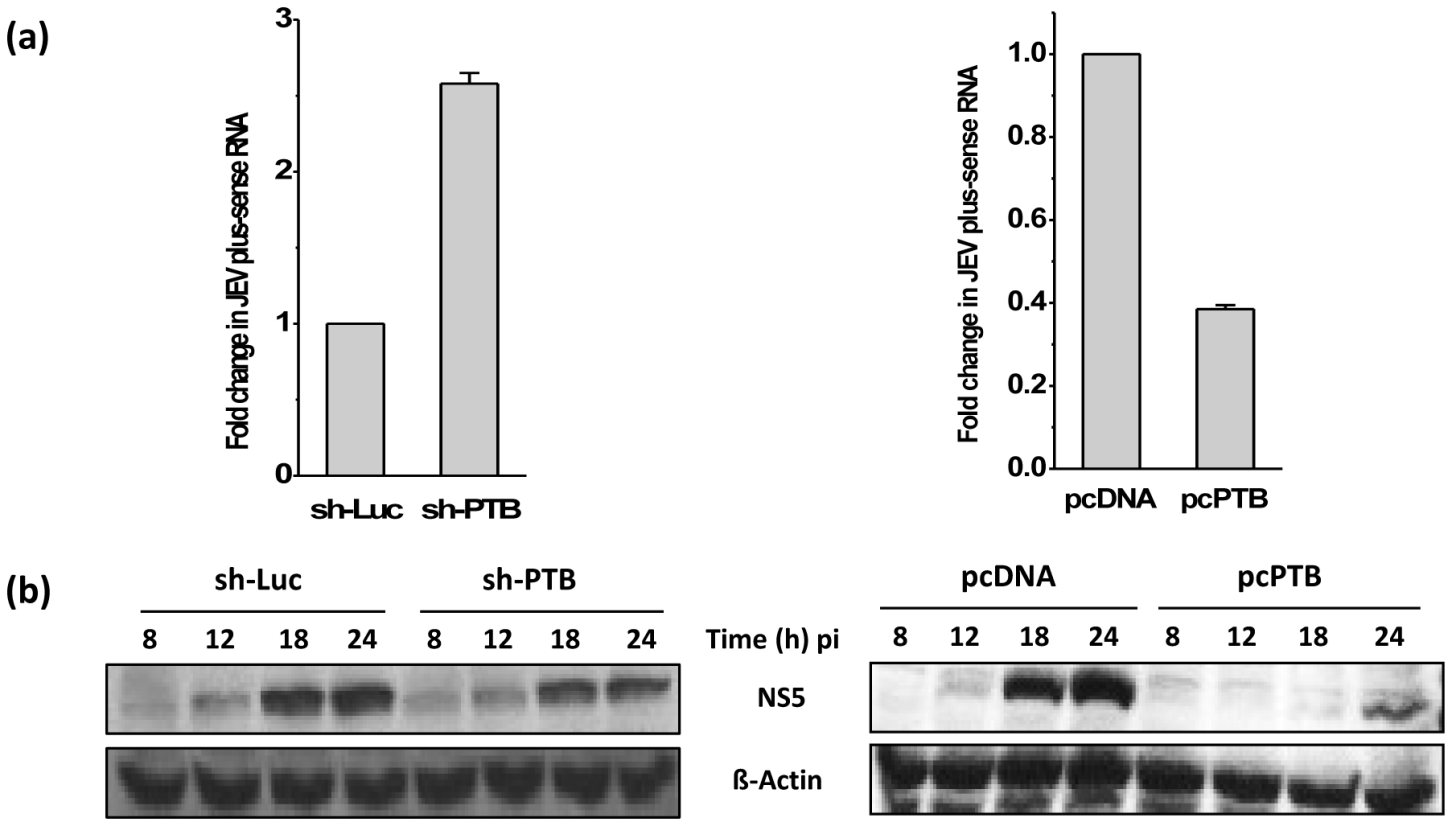
RNA and protein levels in PTB-silenced and over-expressing cells. Vero cells were transfected with sh-Luc or sh-PTB, and pcPTB or pcDNA plasmids and infected 48 h later with JEV at a moi of 1. At indicated time points, cells were harvested to isolate total RNA and proteins. (a) Levels of plus-sense JEV RNA were quantified 24 h pi in PTB-silenced cells (top left panel) or PTB over-expressing cells (top right panel) by real time PCR using 18S rRNA as the internal control. Fold change in JEV RNA was calculated by comparing the RNA levels in PTB-silenced or PTB over-expressing cells with that in the cells transfected with control plasmids sh-Luc or pcDNA, respectively. Mean and standard deviation of data from 3 experiments is shown. (b) The levels of JEV NS5 in cells at various time points were studied by Western blotting. ß-Actin was used as the loading control.

PTB binding to viral RNA could affect both RNA transcription as well as translation that may result in reduced viral titers. We therefore analysed the effect of PTB silencing and over-expression on JEV RNA level by performing quantitative real time RT-PCR for JEV RNA in infected Vero cells. The knock-down of PTB label and its over-expression was established by Western blotting as before. While there was no effect seen on the levels of JEV negative-strand RNA, a ∼150% increase in JEV plus-strand RNA accumulation was observed at 24 h pi in PTB-silenced cells when compared with the control (Fig. 7a). On the other hand, PTB-over-expressing cells showed ∼60% reduction in JEV plus-strand RNA at 24 h pi compared with the control, whereas the levels of JEV negative-strand RNA were not affected. Further, effect of PTB silencing and over-expression on translation of viral proteins was studied by Western blotting. While no differences were seen in JEV NS5 protein levels in PTB knock-down cells, these were significantly decreased in PTB over-expressing cells as compared to the control cells (Fig. 7b). Levels of NS3 protein were affected in a similar fashion. These observations were reproduced in HEK cells also (data not shown).

Enhanced plus-strand RNA levels in PTB knocked-down cells may not necessarily result in enhanced levels of NS5/NS3 if protein synthesis capability of the cell is limiting. On the other hand, reduced levels of plus-strand RNA may result in reduced NS5 accumulation. The data presented above, thus, indicate that PTB plays a negative role in JEV replication by suppressing plus-strand RNA synthesis that in turn could affect the accumulation of viral proteins and viral titers.

### PTB can competitively inhibit NS5 binding to JEV negative-strand RNA

**Figure 8:**
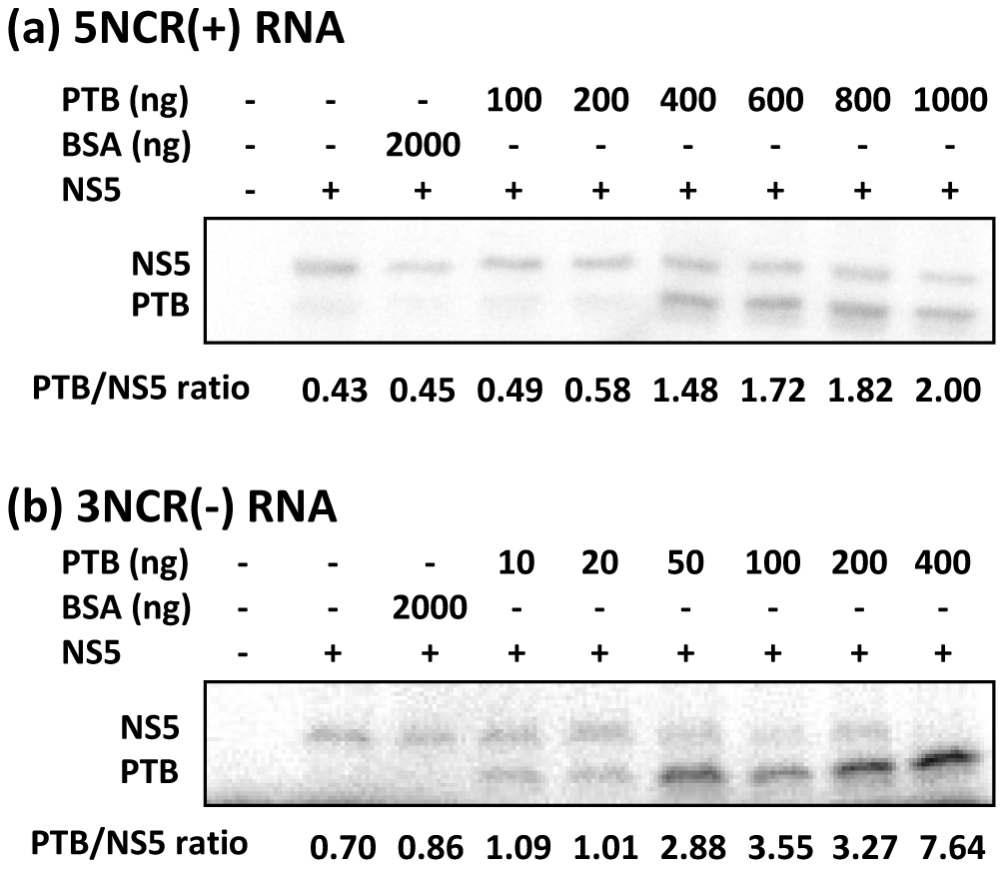
Competitive binding of PTB and NS5 with JEV RNA. RNA-protein binding reaction was carried out using 1 ng of radiolabelled (a) 5 NCR(+) RNA or (b) 3NCR(-) RNA with 600 ng JEV NS5 in the presence of increasing amounts of PTB. The RNA-protein complexes were cross-linked with UV treatment. The RNase A digested product was electrophoresed on a 12% SDS-PAGE followed by autoradiography. Ratio of RNA-bound PTB/NS5, shown at the bottom, has been calculated based on the band intensities.

Association of NS5, the viral replicase protein, with NCRs is necessary for flavivirus replication to occur [40]. We and others have observed by EMSA that JEV NS5 binds to the 5′-NCR of the JEV genome and its complementary sequence in the antigenome. Since PTB was found to strongly interact with both these NCRs *in vitro*, the competitive binding of PTB and NS5 to 5NCR(+) and 3NCR(-) was studied by UV-induced cross-linking assay followed by RNase digestion. A constant amount of NS5 protein and increasing amount of PTB was added in a binding reaction with radiolabelled 5NCR(+) and 3NCR(-) RNA. The UV cross-linked RNA-protein complexes were treated with RNase A and the products were electrophoresed and autoradiographed. As shown in Fig. 8, in the absence of any RNA-binding protein the RNA got completely digested and no band of any complex was seen. In the presence of NS5, a specific RNA-protein complex could be visualized. As the concentration of PTB increased in the binding reaction, the binding of NS5 to 5NCR(+) RNA was not affected. However, binding of NS5 with 3NCR(-) RNA was inhibited with the increasing PTB concentration and it was completely inhibited in presence of high amounts of PTB in the binding reaction. No inhibition of NS5 binding to viral RNAs was observed in presence of BSA. These data indicate that PTB can competitively inhibit NS5 binding to 3NCR(-) RNA which is important for the positive-strand RNA synthesis. These data also confirmed our earlier finding that PTB had higher affinity to 3NCR(-) RNA than to 5NCR(+). This is borne out of the empirical observation that under identical conditions 10 ng PTB showed clear binding to 3NCR(-) whereas 400 ng PTB was needed to show similar binding to 5NCR(+) (Fig. 8).

### Enhanced cAMP levels inhibit JEV replication

**Figure 9:**
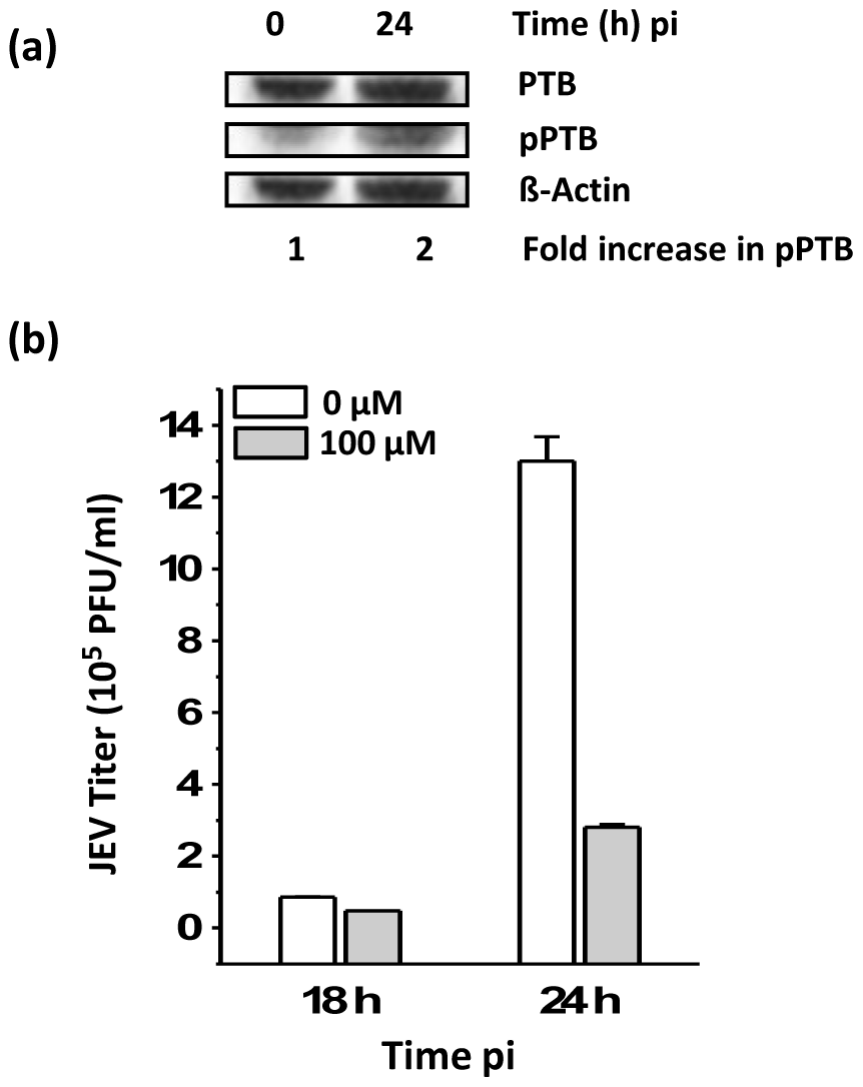
Effect of PTB phosphorylation on JEV replication. (a) Vero cells infected with JEV (moi 1) were lysed at different times pi to prepare total lysate and cytoplasmic faction. These were Western-blotted for the levels of total PTB and phosphorylated-PTB (pPTB) using rabbit phospho-Ser-16 (pS16)-specific antibody (a kind gift from Dr. J. Xie). Fold increase in the levels of pPTB at 24 h were determined by comparing the levels seen at 0 h. ß-Actin was used as the loading control. (b) Vero cells were incubated with 100 µM 8-bromo cAMP and 16 h later infected with JEV at a moi 1. Culture supernatants were collected 18 h and 24 h pi and JEV titers determined. Mean virus titers and standard deviation from 3 experiments are shown.

Nucleo-cytoplasmic transport of PTB is known to be regulated by the cAMP-dependent Protein kinase A (PKA) through direct phosphorylation of PTB [41]. To check whether translocation of PTB during JEV infection is guided by any phosphorylation signal, we studied the phosphorylation level of cytoplasmic PTB in JEV-infected cells. Western blotting using antibody specific for Serine-16 phosphorylated PTB [41] showed a 100% increase in phosho-PTB level in the cytoplasmic fraction of JEV-infected cells although total cellular levels of PTB did not change (Fig. 9a). To further analyse the role of PKA in PTB phosphorylation and translocation to the cytoplasm, Vero cells were treated with a cAMP analogue, 8-bromo cAMP which is converted to cAMP inside the cell and acts as immediate upstream activator of PKA [42]. Following treatment with 8-bromo cAMP, cells were infected with JEV and virus titers determined. The viral titers were 50% reduced at 18 h pi and this reduction was further pronounced to 80% at 24 h pi in cAMP analogue-treated cells (Fig. 9b). These data indicate that increase in cAMP levels can inhibit JEV replication probably through PKA mediated phosphorylation of PTB.

## Discussion

The NCRs of the plus-strand genome of RNA viruses, and their complementary sequences in the antigenome, contain elements important for protein translation and RNA replication. Several host proteins have been described that bind these regions and are exploited by virus for its replication. It could also be envisaged that host defence proteins may bind these NCRs and suppress viral replication by interfering with the RNA translation and/or replication processes. Here we describe a novel antiviral function for PTB during JEV infection where it down modulates genomic RNA synthesis by binding to the 3′-end of the antigenome thereby suppressing JEV replication.

PTB is a 531-amino acid, 58-kDa ubiquitous RNA-binding protein. It was originally identified as a protein with a role in splicing but it is now known to function in a large number of diverse cellular processes including alternative spicing [43], mRNA stabilization [44], [45], polyadenylation [46], [47] and non-traditional translation initiation [48], [49]. The protein contains four RNA-recognition-motif type RNA binding domains. It has been reported to stabilize cellular mRNAs by binding to NCRs for insulin [50], vascular endothelial growth factor [51], CD154 [52], inducible nitric oxide synthase [53], and phosphoglycerate kinase 2 [54]. PTB has also been shown to interact with the genome of several single-stranded positive-sense RNA viruses. For example, PTB interacts with the 5′-NCR of Polio virus genome [55], 5′-leader RNA of Mouse hepatitis virus [56], 3′-NCR of DENV-4 [14] and DENV-2 RNA [15], 5′- and 3′-NCRs of CVB3 RNA [27], and IRES of HCV RNA [57]. PTB was previously shown to bind the JEV 3NCR(-) *in vitro* [29]. Using EMSA and UV-cross linking, here we show that PTB also bound to 5NCR(+) of JEV genome although it had higher affinity for 3NCR(-) RNA. That PTB indeed associated with JEV RNA during viral infection of cell was established by RNA-protein colocalization in infected cells and JEV RNA pull down using PTB antibody. The preferred RNA-binding site of PTB is a U/C tract [58]–[60] and it was shown to interact with a poly U sequence in the 3′-NCR of HCV genomic RNA [61]. A polypyrimidine tract, CUUCUU (nucleotides 20–25 of JEV genome) exists in the 5′ stem of the 5′-NCR which may be involved in interaction with PTB. The JEV 3NCR(-) RNA contains the sequence UCUAA that is thought to be the putative PTB binding site [14], [56], [62].

In picornaviruses, such as polio and CVB3, PTB acts as an IRES *trans*-acting factor activating viral translation initiation [22], [27], [63]. PTB is reported to bind HCV RNA too, although there are conflicting reports on its effects on IRES-mediated translation and virus replication [23], [24], [64]–[66]. To examine the role of PTB in JEV replication the protein levels were down-regulated by siRNA or up-regulated by plasmid-based PTB over-expression in cultured cells where JEV replication was monitored by determining the virus titres or by studying the JEV RNA and protein levels in virus-infected cells. A significant increase in JEV titer in PTB knocked-down cells and a significant suppression of virus titer in PTB over-expressing cells showed that PTB acted as a negative regulator of JEV replication. Our studies show that PTB binds the 3′-end of the negative-sense replication intermediate RNA and competes efficiently with the binding of NS5 to this end of RNA. It may be noted that binding of NS5 to the 3′-end of the negative-sense replication intermediate RNA is required for the synthesis of plus-sense genomic RNA. Indeed, in PTB over-expressing cells we saw significantly reduced levels of plus-sense JEV RNA. This may contribute to suppression of JEV titers in PTB over-expressing cells. Similarly, cells with knocked-down levels of PTB showed increased levels of plus-sense JEV RNA and enhanced viral titers. These results suggest that PTB suppressed JEV replication in cells by inhibiting JEV genomic RNA synthesis. These observations were similar in both Vero and HEK cells and thus negative role of PTB in JEV replication was not cell type specific.

PTB has been shown to negatively regulate translation in the case of Feline calicivirus [67] and HCV [23] although its effect on viral titers is not known. In the case of Transmissible gastroenteritis virus (TGEV), PTB had a negative effect both on viral RNA accumulation and titers [68]. Our observation is strikingly different from that seen in the case of another flavivirus, DENV-2, where PTB knock-down inhibited production of infectious virus and thus PTB was necessary for efficient virus propagation [13]. Interestingly, PTB had no role in YFV replication, another flavivirus [13]. It would, thus, appear that PTB may play varied role in RNA virus replication. Specificity of PTB function may be achieved by its interaction with additional proteins of cellular and viral origin besides its interaction with different RNA elements in different viruses.

PTB is primarily a nuclear protein and to play any role in JEV replication, it should translocate to the cytoplasm. In the present study, PTB was found to translocate to cytoplasm and colocalize with JEV RNA as early as 6 h pi. The translocation of PTB from the nucleus to the cytoplasm has been documented during infection with several RNA viruses, such as poliovirus, rhinovirus, feline calicivirus, and HCV [25], [62], [67]. In case of TGEV, a coronavirus with positive-strand RNA genome, PTB has been shown to colocalize with the viral RNA in the cytoplasm [68]. In case of DENV-2 infection, PTB was shown to translocate to cytoplasm in Vero cells [15] whereas no such translocation was seen in Huh 7 cells [13], [28]. This difference was postulated to be related to the intrinsic differences between the two cells types including the fact that Vero cells did not express interferon stimulated genes [15]. It is important to note, however, that PTB translocated to the cytoplasm following JEV infection of both Vero and HEK cells, and it had inhibitory role in JEV replication in both these cells.

The N-terminal 55-amino acids of PTB contain both nuclear localization signal (NLS) and nuclear export signal (NES) [58], [69]–[71]. Phosphorylation of Ser-16 in the NES is required for accumulation of PTB in the cytoplasm [41]. In our studies, as PTB translocated to the cytoplasm during JEV infection the levels of phosphorylated PTB increased significantly. In addition, enhancement of intracellular levels of cAMP resulted in significantly suppressed JEV titers in Vero cells. In PC-12 cells, nucleo-cytoplasmic shuttling of PTB is regulated by cAMP-dependent protein kinase (PKA) which directly phosphorylates Ser-16 of PTB [41]. These data together suggest that JEV replication may be controlled by the host cells through PTB phosphorylation which is mediated by the cAMP-PKA activation pathway. These are preliminary findings and additional experiments need to be carried out to investigate PTB phosphorylation and its role in JEV replication.

The innate immune response is the first line of defence against foreign pathogens. The recognition of virus-associated molecular patterns, including double- and single-stranded RNA, by pattern recognition receptors initiates a cascade of signalling reactions. These result in the transcriptional up regulation and secretion of pro-inflammatory cytokines that induce an antiviral state. IL-6 and other cytokines are synthesized by cells following the JEV infection [72], [73]. Intracellular cAMP is an important second messenger in several signalling pathways, including IL-6 response [74]–[76]. Our data here show that increased levels of cAMP inhibited JEV replication through PTB translocation and binding to JEV RNA. These data thus present a novel innate defence mechanism by which a host cell may be able to control the virus infection.

## References

[1] World Health Organization (1998) Japanese encephalitis vaccines. Wkly Epidemiol Rec 73:337–344. 9817024

[2] Sumiyoshi H, Mori C, Fuke I, Morita K, Kuhara S, et al (1987) Complete nucleotide sequence of the Japanese encephalitis virus genome RNA. Virology 161:497–510. 3686827 10.1016/0042-6822(87)90144-9

[3] Brinton MA, Dispoto JH (1988) Sequence and secondary structure analysis of the 5'-terminal region of flavivirus genome RNA. Virology 162:290–299. 2829420 10.1016/0042-6822(88)90468-0

[4] Hahn CS, Hahn YS, Rice CM, Lee E, Dalgarno L, et al (1987) Conserved elements in the 3' untranslated region of flavivirus RNAs and potential cyclization sequences. J Mol Biol 198:33–41. 2828633 10.1016/0022-2836(87)90455-4

[5] Gebhard LG, Filomatori CV, Gamarnik AV (2011) Functional RNA elements in the dengue virus genome. Viruses 3:1739–1756. 21994804 10.3390/v3091739 PMC3187688

[6] Yu L, Nomaguchi M, Padmanabhan R, Markoff L (2008) Specific requirements for elements of the 5' and 3' terminal regions in flavivirus RNA synthesis and viral replication. Virology 374:170–185. 18234265 10.1016/j.virol.2007.12.035 PMC3368002

[7] Alvarez DE, Lodeiro MF, Luduena SJ, Pietrasanta LI, Gamarnik AV (2005) Long-range RNA-RNA interactions circularize the dengue virus genome. J Virol 79:6631–6643. 15890901 10.1128/JVI.79.11.6631-6643.2005 PMC1112138

[8] Lodeiro MF, Filomatori CV, Gamarnik AV (2009) Structural and functional studies of the promoter element for dengue virus RNA replication. J Virol 83:993–1008. 19004935 10.1128/JVI.01647-08 PMC2612346

[9] Emara MM, Brinton MA (2007) Interaction of TIA-1/TIAR with West Nile and dengue virus products in infected cells interferes with stress granule formation and processing body assembly. Proc Natl Acad Sci USA 104:9041–9046. 17502609 10.1073/pnas.0703348104 PMC1885624

[10] Vashist S, Anantpadma M, Sharma H, Vrati S (2009) La protein binds the predicted loop structures in the 3' non-coding region of Japanese encephalitis virus genome: role in virus replication. J Gen Virol 90:1343–1352. 19264640 10.1099/vir.0.010850-0

[11] Vashist S, Bhullar D, Vrati S (2011) La protein can simultaneously bind to both 3'- and 5'-noncoding regions of Japanese encephalitis virus genome. DNA Cell Biol 30:339–346. 21294637 10.1089/dna.2010.1114

[12] Isken O, Grassmann CW, Sarisky RT, Kann M, Zhang S, et al (2003) Members of the NF90/NFAR protein group are involved in the life cycle of a positive-strand RNA virus. EMBO J 22:5655–5665. 14592965 10.1093/emboj/cdg562 PMC275419

[13] Anwar A, Leong KM, Ng ML, Chu JJ, Garcia-Blanco MA (2009) The polypyrimidine tract-binding protein is required for efficient dengue virus propagation and associates with the viral replication machinery. J Biol Chem 284:17021–17029. 19380576 10.1074/jbc.M109.006239 PMC2719340

[14] De Nova-Ocampo M, Villegas-Sepulveda N, del Angel RM (2002) Translation elongation factor-1alpha, La, and PTB interact with the 3' untranslated region of dengue 4 virus RNA. Virology 295:337–347. 12033793 10.1006/viro.2002.1407

[15] Agis-Juarez RA, Galvan I, Medina F, Daikoku T, Padmanabhan R, et al (2009) Polypyrimidine tract-binding protein is relocated to the cytoplasm and is required during dengue virus infection in Vero cells. J Gen Virol 90:2893–2901. 19692542 10.1099/vir.0.013433-0

[16] Yi Z, Sperzel L, Nurnberger C, Bredenbeek PJ, Lubick KJ, et al (2011) Identification and characterization of the host protein DNAJC14 as a broadly active flavivirus replication modulator. PLoS Pathog 7:e1001255. 21249176 10.1371/journal.ppat.1001255 PMC3020928

[17] Chien HL, Liao CL, Lin YL (2011) FUSE binding protein 1 interacts with untranslated regions of Japanese encephalitis virus RNA and negatively regulates viral replication. J Virol 85:4698–4706. 21367899 10.1128/JVI.01950-10 PMC3126168

[18] Paranjape SM, Harris E (2007) Y box-binding protein-1 binds to the dengue virus 3'-untranslated region and mediates antiviral effects. J Biol Chem 282:30497–30508. 17726010 10.1074/jbc.M705755200

[19] Ghetti A, Pinol-Roma S, Michael WM, Morandi C, Dreyfuss G (1992) hnRNP I, the polypyrimidine tract-binding protein: distinct nuclear localization and association with hnRNAs. Nucleic Acids Res 20:3671–3678. 1641332 10.1093/nar/20.14.3671 PMC334017

[20] Spriggs KA, Bushell M, Mitchell SA, Willis AE (2005) Internal ribosome entry segment-mediated translation during apoptosis: the role of IRES-trans-acting factors. Cell Death Differ 12:585–591. 15900315 10.1038/sj.cdd.4401642

[21] Spellman R, Smith CW (2006) Novel modes of splicing repression by PTB. Trends Biochem Sci 31:73–76. 16403634 10.1016/j.tibs.2005.12.003

[22] Jang SK (2006) Internal initiation: IRES elements of picornaviruses and hepatitis c virus. Virus Res 119:2–15. 16377015 10.1016/j.virusres.2005.11.003

[23] Tischendorf JJ, Beger C, Korf M, Manns MP, Kruger M (2004) Polypyrimidine tract-binding protein (PTB) inhibits Hepatitis C virus internal ribosome entry site (HCV IRES)-mediated translation, but does not affect HCV replication. Arch Virol 149:1955–1970. 15669107 10.1007/s00705-004-0341-8

[24] Chang KS, Luo G (2006) The polypyrimidine tract-binding protein (PTB) is required for efficient replication of hepatitis C virus (HCV) RNA. Virus Res 115:1–8. 16102869 10.1016/j.virusres.2005.06.012

[25] Florez PM, Sessions OM, Wagner EJ, Gromeier M, Garcia-Blanco MA (2005) The polypyrimidine tract binding protein is required for efficient picornavirus gene expression and propagation. J Virol 79:6172–6179. 15858002 10.1128/JVI.79.10.6172-6179.2005 PMC1091667

[26] Song Y, Tzima E, Ochs K, Bassili G, Trusheim H, et al (2005) Evidence for an RNA chaperone function of polypyrimidine tract-binding protein in picornavirus translation. RNA 11:1809–1824. 16314455 10.1261/rna.7430405 PMC1370870

[27] Verma B, Bhattacharyya S, Das S (2010) Polypyrimidine tract-binding protein interacts with coxsackievirus B3 RNA and influences its translation. J Gen Virol 91:1245–1255. 20071487 10.1099/vir.0.018507-0

[28] Jiang L, Yao H, Duan X, Lu X, Liu Y (2009) Polypyrimidine tract-binding protein influences negative strand RNA synthesis of dengue virus. Biochem Biophys Res Commun 385:187–192. 19450550 10.1016/j.bbrc.2009.05.036 PMC7117538

[29] Kim SM, Jeong YS (2006) Polypyrimidine tract-binding protein interacts with the 3' stem-loop region of Japanese encephalitis virus negative-strand RNA. Virus Res 115:131–140. 16181699 10.1016/j.virusres.2005.07.013

[30] Vrati S, Agarwal V, Malik P, Wani SA, Saini M (1999) Molecular characterization of an Indian isolate of Japanese encephalitis virus that shows an extended lag phase during growth. J Gen Virol 80:1665–1671. 10423134 10.1099/0022-1317-80-7-1665

[31] Ta M, Vrati S (2000) Mov34 protein from mouse brain interacts with the 3' noncoding region of Japanese encephalitis virus. J Virol 74:5108–5115. 10799585 10.1128/jvi.74.11.5108-5115.2000 PMC110863

[32] Huang JL, Lin HT, Wang YM, Weng MH, Ji DD, et al (2004) Sensitive and specific detection of strains of Japanese encephalitis virus using a one-step TaqMan RT-PCR technique. J Med Virol 74:589–596. 15484282 10.1002/jmv.20218 PMC7166820

[33] Welsch S, Miller S, Romero-Brey I, Merz A, Bleck CK, et al (2009) Composition and three-dimensional architecture of the dengue virus replication and assembly sites. Cell Host Microbe 5:365–375. 19380115 10.1016/j.chom.2009.03.007 PMC7103389

[34] Muller DA, Young PR (2013) The flavivirus NS1 protein: molecular and structural biology, immunology, role in pathogenesis and application as a diagnostic biomarker. Antiviral Res 98:192–208. 23523765 10.1016/j.antiviral.2013.03.008

[35] Salonen A, Ahola T, Kaariainen L (2005) Viral RNA replication in association with cellular membranes. Curr Top Microbiol Immunol 285:139–173. 15609503 10.1007/3-540-26764-6_5 PMC7120253

[36] Uchil PD, Satchidanandam V (2003) Architecture of the flaviviral replication complex. Protease, nuclease, and detergents reveal encasement within double-layered membrane compartments. J Biol Chem 278:24388–24398. 12700232 10.1074/jbc.M301717200

[37] Mackenzie JM, Kenney MT, Westaway EG (2007) West Nile virus strain Kunjin NS5 polymerase is a phosphoprotein localized at the cytoplasmic site of viral RNA synthesis. J Gen Virol 88:1163–1168. 17374759 10.1099/vir.0.82552-0

[38] Panyasrivanit M, Khakpoor A, Wikan N, Smith DR (2009) Co-localization of constituents of the dengue virus translation and replication machinery with amphisomes. J Gen Virol 90:448–456. 19141455 10.1099/vir.0.005355-0

[39] Espada-Murao LA, Morita K (2011) Delayed cytosolic exposure of Japanese encephalitis virus double-stranded RNA impedes interferon activation and enhances viral dissemination in porcine cells. J Virol 85:6736–6749. 21525349 10.1128/JVI.00233-11 PMC3126492

[40] Chen CJ, Kuo MD, Chien LJ, Hsu SL, Wang YM, et al (1997) RNA-protein interactions: involvement of NS3, NS5, and 3' noncoding regions of Japanese encephalitis virus genomic RNA. J Virol 71:3466–3473. 9094618 10.1128/jvi.71.5.3466-3473.1997 PMC191493

[41] Xie J, Lee JA, Kress TL, Mowry KL, Black DL (2003) Protein kinase A phosphorylation modulates transport of the polypyrimidine tract-binding protein. Proc Natl Acad Sci USA 100:8776–8781. 12851456 10.1073/pnas.1432696100 PMC166389

[42] Das R, Esposito V, Abu-Abed M, Anand GS, Taylor SS, et al (2007) cAMP activation of PKA defines an ancient signaling mechanism. Proc Natl Acad Sci USA 104:93–98. 17182741 10.1073/pnas.0609033103 PMC1765484

[43] Spellman R, Rideau A, Matlin A, Gooding C, Robinson F, et al (2005) Regulation of alternative splicing by PTB and associated factors. Biochem Soc Trans 33:457–460. 15916540 10.1042/BST0330457

[44] Fred RG, Tillmar L, Welsh N (2006) The role of PTB in insulin mRNA stability control. Curr Diabetes Rev 2:363–366. 18220641 10.2174/157339906777950570

[45] Matus-Nicodemos R, Vavassori S, Castro-Faix M, Valentin-Acevedo A, Singh K, et al (2011) Polypyrimidine tract-binding protein is critical for the turnover and subcellular distribution of CD40 ligand mRNA in CD4+ T cells. J Immunol 186:2164–2171. 21242519 10.4049/jimmunol.1003236 PMC3119477

[46] Castelo-Branco P, Furger A, Wollerton M, Smith C, Moreira A, et al (2004) Polypyrimidine tract binding protein modulates efficiency of polyadenylation. Mol Cell Biol 24:4174–4183. 15121839 10.1128/MCB.24.10.4174-4183.2004 PMC400487

[47] Millevoi S, Decorsiere A, Loulergue C, Iacovoni J, Bernat S, et al (2009) A physical and functional link between splicing factors promotes pre-mRNA 3' end processing. Nucleic Acids Res 37:4672–4683. 19506027 10.1093/nar/gkp470 PMC2724285

[48] Niepmann M, Petersen A, Meyer K, Beck E (1997) Functional involvement of polypyrimidine tract-binding protein in translation initiation complexes with the internal ribosome entry site of foot-and-mouth disease virus. J Virol 71:8330–8339. 9343186 10.1128/jvi.71.11.8330-8339.1997 PMC192292

[49] Borman A, Howell MT, Patton JG, Jackson RJ (1993) The involvement of a spliceosome component in internal initiation of human rhinovirus RNA translation. J Gen Virol 74:1775–1788. 8397279 10.1099/0022-1317-74-9-1775

[50] Tillmar L, Carlsson C, Welsh N (2002) Control of insulin mRNA stability in rat pancreatic islets. Regulatory role of a 3'-untranslated region pyrimidine-rich sequence. J Biol Chem 277:1099–1106. 11696543 10.1074/jbc.M108340200

[51] Coles LS, Bartley MA, Bert A, Hunter J, Polyak S, et al (2004) A multi-protein complex containing cold shock domain (Y-box) and polypyrimidine tract binding proteins forms on the vascular endothelial growth factor mRNA. Potential role in mRNA stabilization. Eur J Biochem 271:648–660. 14728692 10.1111/j.1432-1033.2003.03968.x

[52] Hamilton BJ, Genin A, Cron RQ, Rigby WF (2003) Delineation of a novel pathway that regulates CD154 (CD40 ligand) expression. Mol Cell Biol 23:510–525. 12509450 10.1128/MCB.23.2.510-525.2003 PMC151525

[53] Pautz A, Linker K, Hubrich T, Korhonen R, Altenhofer S, et al (2006) The polypyrimidine tract-binding protein (PTB) is involved in the post-transcriptional regulation of human inducible nitric oxide synthase expression. J Biol Chem 281:32294–32302. 16950790 10.1074/jbc.M603915200

[54] Xu M, Hecht NB (2007) Polypyrimidine tract binding protein 2 stabilizes phosphoglycerate kinase 2 mRNA in murine male germ cells by binding to its 3'UTR. Biol Reprod 76:1025–1033. 17329592 10.1095/biolreprod.107.060079

[55] Gutierrez AL, Denova-Ocampo M, Racaniello VR, del Angel RM (1997) Attenuating mutations in the poliovirus 5' untranslated region alter its interaction with polypyrimidine tract-binding protein. J Virol 71:3826–3833. 9094658 10.1128/jvi.71.5.3826-3833.1997 PMC191533

[56] Li HP, Huang P, Park S, Lai MM (1999) Polypyrimidine tract-binding protein binds to the leader RNA of mouse hepatitis virus and serves as a regulator of viral transcription. J Virol 73:772–777. 9847386 10.1128/jvi.73.1.772-777.1999 PMC103887

[57] Ali N, Siddiqui A (1995) Interaction of polypyrimidine tract-binding protein with the 5' noncoding region of the hepatitis C virus RNA genome and its functional requirement in internal initiation of translation. J Virol 69:6367–6375. 7666538 10.1128/jvi.69.10.6367-6375.1995 PMC189536

[58] Perez I, Lin CH, McAfee JG, Patton JG (1997) Mutation of PTB binding sites causes misregulation of alternative 3' splice site selection in vivo. RNA 3:764–778. 9214659 PMC1369523

[59] Sawicka K, Bushell M, Spriggs KA, Willis AE (2008) Polypyrimidine-tract-binding protein: a multifunctional RNA-binding protein. Biochem Soc Trans 36:641–647. 18631133 10.1042/BST0360641

[60] Singh R, Valcarcel J, Green MR (1995) Distinct binding specificities and functions of higher eukaryotic polypyrimidine tract-binding proteins. Science 268:1173–1176. 7761834 10.1126/science.7761834

[61] Luo G (1999) Cellular proteins bind to the poly(U) tract of the 3' untranslated region of hepatitis C virus RNA genome. Virology 256:105–118. 10087231 10.1006/viro.1999.9639

[62] Choi KS, Huang P, Lai MM (2002) Polypyrimidine-tract-binding protein affects transcription but not translation of mouse hepatitis virus RNA. Virology 303:58–68. 12482658 10.1006/viro.2002.1675

[63] Martinez-Salas E, Pacheco A, Serrano P, Fernandez N (2008) New insights into internal ribosome entry site elements relevant for viral gene expression. J Gen Virol 89:611–626. 18272751 10.1099/vir.0.83426-0

[64] Domitrovich AM, Diebel KW, Ali N, Sarker S, Siddiqui A (2005) Role of La autoantigen and polypyrimidine tract-binding protein in HCV replication. Virology 335:72–86. 15823607 10.1016/j.virol.2005.02.009

[65] Aizaki H, Choi KS, Liu M, Li YJ, Lai MM (2006) Polypyrimidine-tract-binding protein is a component of the HCV RNA replication complex and necessary for RNA synthesis. J Biomed Sci 13:469–480. 16691359 10.1007/s11373-006-9088-4

[66] Ito T, Lai MM (1999) An internal polypyrimidine-tract-binding protein-binding site in the hepatitis C virus RNA attenuates translation, which is relieved by the 3'-untranslated sequence. Virology 254:288–296. 9986795 10.1006/viro.1998.9541

[67] Karakasiliotis I, Vashist S, Bailey D, Abente EJ, Green KY, et al (2010) Polypyrimidine tract binding protein functions as a negative regulator of feline calicivirus translation. PLoS One 5:e9562. 20224775 10.1371/journal.pone.0009562 PMC2835748

[68] Sola I, Galan C, Mateos-Gomez PA, Palacio L, Zuniga S, et al (2011) The polypyrimidine tract-binding protein affects coronavirus RNA accumulation levels and relocalizes viral RNAs to novel cytoplasmic domains different from replication-transcription sites. J Virol 85:5136–5149. 21411518 10.1128/JVI.00195-11 PMC3126201

[69] Romanelli MG, Morandi C (2002) Importin alpha binds to an unusual bipartite nuclear localization signal in the heterogeneous ribonucleoprotein type I. Eur J Biochem 269:2727–2734. 12047381 10.1046/j.1432-1033.2002.02942.x

[70] Li B, Yen TS (2002) Characterization of the nuclear export signal of polypyrimidine tract-binding protein. J Biol Chem 277:10306–10314. 11781313 10.1074/jbc.M109686200

[71] Kamath RV, Leary DJ, Huang S (2001) Nucleocytoplasmic shuttling of polypyrimidine tract-binding protein is uncoupled from RNA export. Mol Biol Cell 12:3808–3820. 11739782 10.1091/mbc.12.12.3808 PMC60757

[72] Sooryanarain H, Sapkal GN, Gore MM (2012) Pathogenic and vaccine strains of Japanese encephalitis virus elicit different levels of human macrophage effector functions. Arch Virol 157:1905–1918. 22729616 10.1007/s00705-012-1386-8

[73] Nazmi A, Dutta K, Basu A (2011) RIG-I mediates innate immune response in mouse neurons following Japanese encephalitis virus infection. PLoS One 6:e21761. 21738791 10.1371/journal.pone.0021761 PMC3128083

[74] Yadav M, Roach SK, Schorey JS (2004) Increased mitogen-activated protein kinase activity and TNF-alpha production associated with Mycobacterium smegmatis- but not Mycobacterium avium-infected macrophages requires prolonged stimulation of the calmodulin/calmodulin kinase and cyclic AMP/protein kinase A pathways. J Immunol 172:5588–5597. 15100302 10.4049/jimmunol.172.9.5588

[75] Chio CC, Chang YH, Hsu YW, Chi KH, Lin WW (2004) PKA-dependent activation of PKC, p38 MAPK and IKK in macrophage: implication in the induction of inducible nitric oxide synthase and interleukin-6 by dibutyryl cAMP. Cell Signal 16:565–575. 14751542 10.1016/j.cellsig.2003.10.003

[76] Zhang Y, Mahendran R, Yap LL, Esuvaranathan K, Khoo HE (2002) The signalling pathway for BCG-induced interleukin-6 production in human bladder cancer cells. Biochem Pharmacol 63:273–282. 11841803 10.1016/s0006-2952(01)00831-0

